# De novo transcriptome reconstruction in aquacultured early life stages of the cephalopod *Octopus vulgaris*

**DOI:** 10.1038/s41597-022-01735-2

**Published:** 2022-10-08

**Authors:** María Prado-Álvarez, Sonia Dios, Pablo García-Fernández, Ricardo Tur, Ismael Hachero-Cruzado, Pedro Domingues, Eduardo Almansa, Inmaculada Varó, Camino Gestal

**Affiliations:** 1 Instituto de Investigaciones Marinas (IIM), CSIC, Eduardo Cabello 6, 36208 Vigo, Spain; 2 Centro Oceanográfico de Vigo (COV-IEO), CSIC, Subida a Radio Faro 50-52, 36390 Vigo, Spain; 3 Centro Oceanográfico de Canarias (COC-IEO), CSIC. Calle La Farola del Mar n° 22, Dársena Pesquera, 38180 Santa Cruz de Tenerife, Spain; 4 Instituto de Acuicultura de Torre de la Sal (IATS), CSIC. Torre de la Sal s/n, 12595 Ribera de Cabanes, Spain; 5 Present Address: Pescanova Biomarine Center, Lugar Ardia 172, 36980 O Grove, Spain

**Keywords:** Databases, Transcriptomics

## Abstract

Cephalopods have been considered enigmatic animals that have attracted the attention of scientists from different areas of expertise. However, there are still many questions to elucidate the way of life of these invertebrates. The aim of this study is to construct a reference transcriptome in *Octopus vulgaris* early life stages to enrich existing databases and provide a new dataset that can be reused by other researchers in the field. For that, samples from different developmental stages were combined including embryos, newly-hatched paralarvae, and paralarvae of 10, 20 and 40 days post-hatching. Additionally, different dietary and rearing conditions and pathogenic infections were tested. At least three biological replicates were analysed per condition and submitted to RNA-seq analysis. All sequencing reads from experimental conditions were combined in a single dataset to generate a reference transcriptome assembly that was functionally annotated. The number of reads aligned to this reference was counted to estimate the transcript abundance in each sample. This dataset compiled a complete reference for future transcriptomic studies in *O. vulgaris*.

## Abstract (summary)

**Table:** 

Measurement(s)	Transcriptome sequencing assay
Technology Type(s)	RNA-seq assay (Illumina)
Sample Characteristic - Organism	*Octopus vulgaris*
Sample Characteristic - Environment	Ocean
Sample Characteristic - Location	NW Spain (Ría de Vigo, Galicia)

## Background & Summary

For decades, cephalopods have attracted the attention of scientists from a wide spectrum of areas of expertise from physiology and neurobiology to animal behaviour and ecology. They are characterised by features of their biology and physiology, which are novel in design and evolutionary adaptation making them interesting models for research^1–7^. They have been considered an enigmatic group of animals that have evolved until acquiring very high capacities, which in turn might be the success of their evolution^8–13^. Octopus intelligence, appearance and the ability to learn, play and regenerate their damaged tissues have ever fascinated. Indeed, cephalopods are the sole invertebrates included in the list of regulated species by the EU directive on the “protection of animals for scientific purposes”^14,15^. Despite recent and relevant advances, there are still many question marks to decipher the way of life, the feeding and reproduction procedures, the habitat and also the potential of one of the smartest group of invertebrates on earth.

The common octopus, *Octopus vulgaris*, inhabits two different habitats depending on the life stage. From hatching to approximately 30–40 days, animals in paralarvae stage compose part of the zooplankton with a high predatory activity^16^. After this time, animals change progressively their habitat and behaviours to a benthonic life in a process called settlement. On settlement, a number of changes in morphology occur including a positive allometric arm growth, new chromatophore, iridophore and leucophore genesis, skin sculptural development and horizontal pupillary response^17–20^. Moreover, after settlement animals loose Kölliker organs, the lateral line system and the oral denticles of beaks and acquire a strong negative phototaxis^17,21,22^. Among the peculiarities of paralarvae stage, it is remarkable that the central nervous system is comparatively a 20% larger in paralarvae than in adults representing the 25% of fresh paralarvae weight^4,17,23^. However, longitudinal and radial musculature, the buccal lateral lobes, the digestive and branchial glands, and the renal appendages (among others) are less developed or simpler in paralarvae than those in adults^19^.

Besides the interest in the developmental stages from embryos to adults in areas such as evolutionary developmental biology (evo-devo) or marine ecology, *O. vulgaris* is highly appreciated for human consumption with an elevated market price^24^. Due to the increased demand on marine products as protein supply, this species was postulated as a good candidate for aquaculture diversification^25–27^. Contrary to other marine species whose commercial culture have been successfully achieved, the octopus rearing remains unsolved due to an important lack of information regarding appropriate environmental parameters and nutritional requirements to assure a correct development and proper establishment in the benthic media^25,28–32^.

Although the molecular basis that regulate the biological processes in cephalopods are not well understood, recent advances in next generation sequencing have allowed the generation of databases including the genome sequencing of four species, namely *O. bimaculoides*^8^, *Callistoctopus minor*^33^ and more recently *Euphrymna scolopes*^34^ and *O. vulgaris*^35^. Due to the high scientific interest and high commercial value of the common octopus, *O. vulgaris*, several transcriptomic studies have been published, including adult specific organs^36,37^ and also paralarvae under different growth conditions^38^. However, the possibilities of transcriptomic studies rely mainly in a good reference for comparison especially in non-model organisms, on which numerous limitations exist such as absence of cell lines or even the maintenance of animals under captivity. The transcriptomic sequencing of all the different conditions presented here will allow future comparative transcriptomic analysis improving the successful transcript annotation in public databases and would also help the optimization and improvement of the current genome ensemble and annotation.

In order to obtain a broad overview of the transcripts expressed under different conditions, several live preys’ diets based on crustacean (*Artemia*, decapods and amphipods) and different rearing conditions (volume and light) were compared along the development of paralarvae from newly hatchlings to 10, 20 and 40 days post-hatching (dph), when pre-settlement behaviour begins. Pathogenic experimental infections were also carried out to complete the present study with an immune response assessment. The bacterium *Vibrio lentus*, associated with mortalities in adult *O. vulgaris*^39^ and the ostreid herpes virus OsHV-1 µVar that has caused mass mortalities in the oyster *Crassostrea gigas*, and recently found in different life stages in *O. vulgaris*^40^ were also included for these assays.

Overall, in this study we have compiled different early life stages of development of the common octopus from the embryo stage to settlement in the benthic media under different conditions to assemble a transcriptome to be used as reference in future studies.

## Methods

### Paralarvae rearing and culture

*O. vulgaris* embryos and paralarvae were obtained from a broodstock comprised by individuals of similar sizes maintained in a 400 L flow-through system tank according to Iglesias *et al*.^25,41^. PVC shelters were provided as refuges to induce natural spawning. Individuals were maintained under standard conditions of summer natural photoperiod (42°11′01″N 8°48′46″O), sea water temperatures (19–23 °C) and they were fed *ad libitum* with thawed crabs and fish three days a week. Once a laying occurs, the female was kept in a separate tank at the same water temperature. The female took care of the spawn without being fed until hatching. Paralarvae were reared following an adaptation of the rearing protocol patented by the Spanish Institute of Oceanography^42^, in black cylindrical 500 L tanks with central aeration, at an initial density of 4 individuals/L. The rearing seawater temperature was 20.8 ± 1.1 °C, oxygen concentration 6.0 ± 0.5 ppm and 35.0 ± 1.0 psu salinity. Light intensity was of 600 lx with a 14 L:10D photoperiod. The flow-through seawater system was equipped with 10, 5 and 1 μm filter cartridges. *Nannochloropsis sp* and *Isochrysis aff. galbana* (T-Iso) were used as green water at 3·10^5^ cells/mL in a 3:1 proportion.

**Fig. 1:**
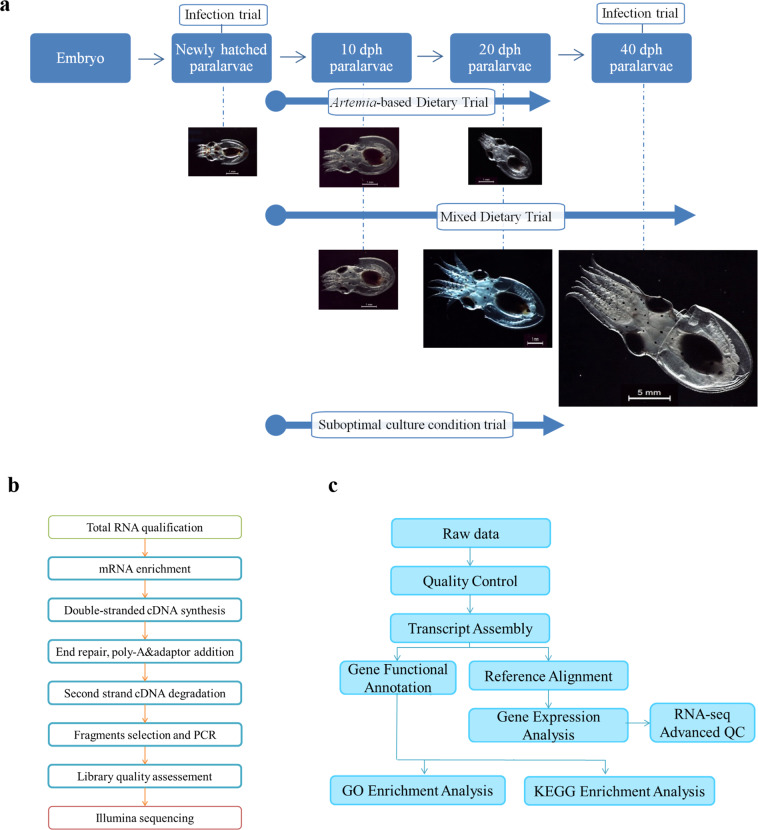
Experimental and RNA-seq workflows of *O. vulgaris* transcriptomes. (**a**) Set of samples collected over the pre-settlement development of *O. vulgaris* (filled coloured boxes) and the three trials (infection, suboptimal culture condition and dietary) carried out at different stages (empty coloured boxes). Pictures of each stage are shown at the corresponding life stage and dietary treatment. Age of paralarvae is indicated in days post-hatching (dph). (**b**) Sample preparation and library construction workflow. (**c**) Transcriptome analysis workflow including construction of reference transcriptome. Photographs by R. Tur.

A set of 20 paralarvae sampled every 10 days from each experimental culture was also used to determine the individual dry weight after oven drying for 24 h at 80 °C^25^. The percentage of the specific growth rate (SGR %) for each trial was calculated following the formula: (% BW/day) = [(LnDW_f_ - LnDW_i_) × 100/(tf-ti)], where BW is the body weight, DWf and DWi are the paralarvae dry weight at final time (tf) and initial time (ti), respectively. The different experimental designs, paralarvae age at collection time and pictures of animals are shown in Fig. 1a.

### Dietary experimental trial

Two dietary treatments were tested on newly hatched paralarvae. *Artemia*-based diet (A) consisted of *Artemia* metanauplii (Sep-Art EG, INVE Aquaculture, Belgium) on-growing for 5–7 days (average size 1.5 mm) with *Tetraselmis chuii* plus T-Iso (50:50) and fed on T-Iso for the last 24 h. Paralarvae were fed *ad libitum* and the *Artemia* concentration adjusted according to the observed consumption. The concentration of *Artemia* supplied per culture tank varied daily between 0.14 and 2.25 µg dry weight/mL, which represents a daily density of 0.03–0.5 preys/mL. The improved mixed diet (M) consisted on four live preys supplied to the culture tank at the following concentration per day: zoeae of spider crab (*Maja brachydactyla*) at 0.20–0.99 µg dry weight/mL (0.002–0.01 preys/mL), zoeae of velvet crab (*Necora puber)* at 0.02–0.12 µg dry weight/mL (0.001–0.005 preys/mL), juveniles of amphipods gammarids (*Jassa* sp.) at 0.08–0.17 µg dry weight/mL (0.0005–0.001 preys/mL) and *Artemia* at 0.23–0.9 µg dry weight/mL (0.05–0.2 preys/mL). Paralarvae fed on A diet were grown until 30 dph, the time point from which the culture survival began to decline, whereas paralarvae fed on M diet showed pre-settlement behaviour at 40 dph. *Artemia* group at 20 dph showed 1.2 ± 0.2 mg of dry weight (SGR: 7.1%), whereas mixed group reached 2.7 ± 0.3 mg (11.12%) at 20 dph and 5.0 ± 1.1 mg (9.5%) at 30 dph.

### Experimental trial under suboptimal rearing conditions

A suboptimal culture condition of octopus paralarvae was evaluated in order to analyse the effect of the stress caused by abiotic factors in animal culture and growth. Paralarvae were reared following the general culture conditions described above^25,41,42^. In order to perform suboptimal conditions, the volume of the culture tanks was reduced to 100 L and light intensity was increased to 1100 lux. The dietary treatment administered was the same as in M diet corresponding to the dietary experimental trial. Paralarvae grew until 20 dph, the time point from which the culture survival began to decline. Suboptimal group of animals reached 1.6 ± 0.2 mg of dry weight (SGR: 8.6%).

### Infection experimental trial

A small number of paralarvae of 0 and 40 dph fed on mixed M diet were exposed *in vivo* to the Gram negative bacteria *V. lentus* and an additional small number of paralarvae of 0 dph were exposed to the virus OsHV-1. A strain of *V. lentus* (kindly provided by Dr. R. Farto, U. Vigo) was maintained frozen at −80 °C until use. After defrosting for a few minutes in ice, bacteria were grown in liquid medium (TSB, Tryptic Soy Broth with supplement of NaCl 2%) by incubation in continuous shaking at 22 °C for 48 h. Bacteria cultures were centrifuged (3000 g) for 3 min, then the medium was discarded and the pellet was suspended in 0.2 µm filtered sea water (FSW) in order to prepare a bacterial suspension to be used for experimental infections. The number of colonies forming units (CFU) was estimated by seeding serial dilutions on TSA medium.

OsHV-1 viral suspension was prepared following Prado-Alvarez *et al*.^40^. Briefly, infected oyster tissues (gill and mantle) were mashed, diluted in artificial sea water (ASW) and filtered under sterilize conditions. Infection on tissues was previously corroborated by conventional PCR. Viral load was quantified by qPCR^43,44^ using a collection of plasmid standards with a known amount of viral load.

Experimental infections were carried out by bath immersion at 22 °C in 6-well plates with 14 paralarvae, each in 12 mL final volume containing 10^8^ CFU/mL of *V. lentus* and 2.6 10^4^ viral copies/µL of OsHV-1 µVar. Control paralarvae were exposed to ASW. Three paralarvae per condition were collected at 1, 4 and 24 hours post-exposure for RNA-seq analysis and a total of 5 additional paralarvae were collected for transmission electron microscopy analysis (TEM).

### Sample collection

For all developmental stages or experimental conditions and before any processing, paralarvae and embryo were anaesthetised using cold sea water (lower than 2 °C), rinsed in distilled water and immersed in RNAlater (QIAGEN, GmbH, Germany) following manufacturer’s instructions for fixation and preservation at −80 °C until use.

In the case of embryo samples, a number of 20 embryos (5 embryos/stage) at stages VIII, X, XV and XVIII (according to Naef, 1928^45^) were sampled from the female shelter and pooled together. Prior to introduction into RNAlater, embryos were anaesthetised as indicated above and punctured in the chorion with a 0.5 × 16 mm needle to ensure proper penetration of the preservative into the embryo. In addition, 5 biological replicates of 1 individual paralarvae each were sampled at 0, after observation of the fourth sucker in the arms (10 dph approximately) and at 20 and 40 dph from the improved mixed diet (M) treatment. In the *Artemia*-based dietary experimental trial and suboptimal culture conditions 5 biological replicates (1 paralarvae each one) were sampled at 20 dph. Finally, in infection experimental trials, 3 biological replicates (1 individual paralarvae each one) of newly hatched paralarvae and also 40 dph paralarvae were sampled at 1, 4 and 24 h post-exposure to *V. lentus*, and similar sampling procedure was performed after exposure to OsHV-1 at 0 dph.

All animal experiments were performed according to the Spanish law RD53/2013 within the framework of European Union directive on animal welfare (Directive 2010/63/EU) for the protection of animals employed for experimentation and other scientific purposes, following the Guidelines for the care and welfare of cephalopods published by Fiorito *et al*.^14^, and approved by the Ethic Committee of the National Competent Authority (project number: CEIBA 2017-0249 for culture experiments, and project number: CEIBA2014-0108; ES360570202001/17/EDUC FORM 07/CGM01 for infection experiments).

### RNA isolation

Total RNA was extracted for both pooled embryos and individual paralarvae using Trizol (Thermo Fisher Scientific®, Waltman, MA, USA) following the manufacturer’s instructions.

### Library preparation and Illumina sequencing

Sequencing libraries were generated using NEBNext® Ultra™ RNA Library Prep Kit for Illumina® (NEB, USA) (Fig. 1b). Index codes were added to attribute sequences to each sample. Briefly, mRNA was purified from total RNA (1 µg) using poly-T oligo-attached magnetic beads. Fragmentation was carried out using divalent cations under elevated temperature in NEBNext First Strand Synthesis Reaction Buffer (5x). First strand cDNA was synthesized using random hexamer primer and M-MuLV Reverse Transcriptase (RNase H). Second strand cDNA synthesis was subsequently performed using DNA Polymerase I and RNase H. In the reaction buffer, dNTPs with dTTP were replaced by dUTP. Remaining overhangs were converted into blunt ends via exonuclease/polymerase activities. After adenylation of 3′ ends of DNA fragments, NEBNext Adaptor with hairpin loop structure were ligated to prepare for hybridization. In order to select cDNA fragments of preferentially 250~300 bp in length, the library fragments were purified with AMPure XP system (Beckman Coulter, Beverly, USA). Then 3 µL USER Enzyme (NEB, USA) was used with size-selected, adaptor-ligated cDNA at 37 °C for 15 min followed by 5 min at 95 °C before PCR. PCR was performed with Phusion High-Fidelity DNA polymerase, Universal PCR primers and Index Primer. Finally, products were purified (AMPure XP system) and library quality was assessed on the Agilent Bioanalyzer 2100 system.

The clustering of the index-coded samples was performed on a cBot Cluster Generation System using PE Cluster Kit cBot-HS (Illumina) according to the manufacturer’s instructions. After cluster generation, the library preparations were sequenced on the Illumina platform Novaseq, where non-stranded and paired-end reads were generated. A total of 46 libraries were constructed and sequenced (Supplementary File 1): 21 libraries, including one pool of different developmental stages of embryos (EMB) and 5 biological replicates of paralarvae fed on improved mixed diet and sampled at different stages: after hatching (OP_0), after observation of the fourth sucker in the arms at approximately 10 dph (OPM_4s) and at 20 and 40 dph (OPM_20 and OPM_40); 5 libraries of 20 dph paralarvae fed on *Artemia*-based diet (OPA_20); 5 libraries corresponding to the suboptimal culture conditions at 20 dph (OS_20); and 15 libraries corresponding to the infection trials: 3 biological replicates per infection condition (24 hours post-exposure) including *V. lentus*, viral OsHV-1 infection and control at 0 dph (VL_0, OsHV_0, C_0) and *V. lentus* and control at 40 dph (VL_40 and C_40). Subsequent bioinformatic analyses are represented in Fig. 1c and described in the following subsections.

### Raw reads quality control

Clean data were obtained by removing reads with adaptor contamination and/or index codes, reads containing uncertain nucleotides in a proportion higher than 10% (N > 10%), and low quality reads when nucleotides with base quality less than 20 constituted more than 50 percent of the read. The error rate (e) for each base was transformed using Phred score (Q_phred_ = −10log_10_(e)). The relationship between Phred score and base quality was established with the Illumina CASAVA v1.8 software. Simultaneously, Q20, Q30, GC content and sequence duplication level of the clean data were calculated.

### Transcriptome reconstruction and hierarchical clustering

Clean reads from the 46 libraries were assembled in a global reference transcriptome. The left files (read1 files) from all libraries/samples were pooled into one big left.fq file, and right files (read2 files) into one big right.fq file. Transcriptome assembly was accomplished based on the left.fq and right.fq using Trinity software^46^ with min_kmer_cov set to 2 and all other parameters set by default. Assembled contigs were clustered following Corset^47^ based on shared reads. The parameters use for each tool and program utilized for bioinformatic analysis are detailed in the Supplementary File 1.

### Transcript functional annotation

Gene function was annotated based on the following databases: Nr (NCBI non-redundant protein sequences), Nt (NCBI non-redundant nucleotide sequences), Pfam (Protein family), KOG/COG (Clusters of Orthologous Groups of proteins), Swiss-Prot (A manually annotated and reviewed protein sequence database), KO (KEGG Orthology database), and GO (Gene Ontology). The software and parameters used in each database were as follows: Nt with NCBI blast 2.2.28 + (*E-*value threshold of 10^−5^); Nr, Swiss-Prot and KOG using Diamond 0.8.22. For Nr and Swiss-Prot databases, the *E*-value threshold was 10^−5^ and 10^−3^ for KOG; the prediction of protein structure domain (Pfam) was evaluated with HMMER 3.0 package (*E*-value threshold of 0.01), and based on the protein annotation results of Nr and Pfam, the GO annotation was carried out using Blast2GO v2.5^48^ (*E*-value threshold of 10^−6^). Finally, KEGG Automatic Annotation Server was used to form the KEGG annotation (*E*-value threshold of 10^−10^).

### Quantification of gene expression levels

Gene expression levels were estimated by RSEM^49^ for each sample using as reference the global *de novo* transcriptome filtered by Corset. Briefly, clean data from each library were mapped back onto the assembled transcriptome using the aligner software Bowtie^50^ to obtain the readcounts for each unigene after mapping. Gene expression level was transformed into FPKM (Fragments Per Kilobase of transcript sequence per Millions base pairs sequenced) values. The parameters utilized for each analysis are indicated in the Supplementary File 1.

## Data Records

Raw read sequencing data were deposited in the NCBI SRA database with accession number PRJNA754143^51^. The transcriptome Shotgun Assembly project has been deposited at DDBJ/EMBL/GenBank under the accession GKAX00000000^52^. Functional annotation of transcripts against Nr (NCBI non-redundant protein sequences), Nt (NCBI non-redundant nucleotide sequences), Pfam (Protein family), KOG/COG (Clusters of Orthologous Groups of proteins), Swiss-Prot (A manually annotated and reviewed protein sequence database), KO (KEGG Orthology database), and GO (Gene Ontology) databases were deposited in figshare^53^ (10.6084/m9.figshare.16685068).

## Technical Validation

### RNA integrity and quality

**Fig. 2:**
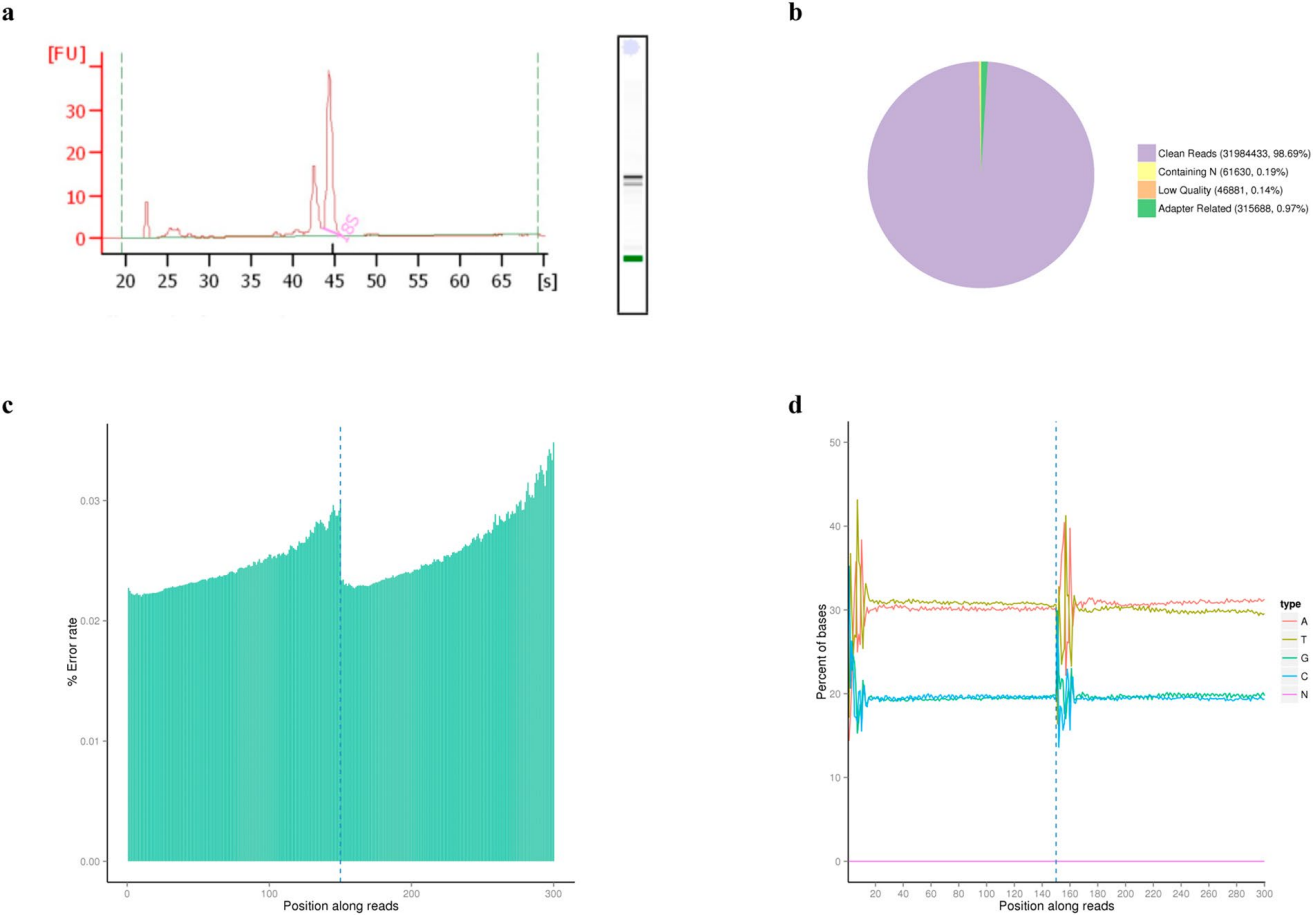
Quality data filtering in a representative sample. (**a**) Electropherogram showing the fluorescence and running time. (**b**) Classification of raw reads into clean reads (purple), reads containing adapter contamination (green), reads containing uncertain nucleotides in more than 10% of the read length (orange) and reads containing uncertain base pairs (N) (yellow). (**c**) Error rate per base position. (**d**) GC content distribution per base position.

A preliminary quantitation and RNA purity was checked using the NanoPhotometer® spectrophotometer (IMPLEN, CA, USA). RNA degradation and potential contamination was assayed by agarose (1%) gel electrophoresis. Finally, quantity and integrity of total RNA was confirmed using the RNA Nano 6000 Assay Kit of the Agilent Bioanalyzer 2100 system (Agilent Technologies, CA, USA). The RNA integrity numbers (RIN) ranged between 6.1 and 8.9. The electropherogram of a representative sample is shown in Fig. 2a. The RIN parameter is not a good indicator of RNA integrity in molluscs due to the 28 S hidden breaks, which leads to two fractions and a different electrophoresis profile compared to model organisms^54,55^. However, this analysis allowed us the detection of degradation in the RNA sample. As it is observed in the representative sample, no degradation was observed in any of the RNA samples obtaining an appropriate quality for subsequent analysis.

### Data filtering

Raw reads were filtered to remove reads containing adapters and reads of low quality. Reads with adaptor contamination were discarded. The acceptable percentage of uncertain nucleotides were set at 10%, therefore reads with higher values were also discarded. Moreover, an extra filtering parameter was considered based on the proportion of the positions containing base quality Phred score below 20. Reads with more than 50% of the positions containing a Phred score below 20 were also eliminated from the analysis. After filtering an average of 97.9% of the raw reads was retained. The sample with lowest retention of clean reads reached also a high percentage of cleaned reads (96.08%) (Supplementary File 1). As an example, the classification of reads in one-end sequencing read of the representative sample resulted in a total of 0.4 M reads removed, including 0.3 M reads filtered due to adaptor contamination, 0.04 M reads with low quality and 0.06 M reads containing uncertain nucleotides (N) in a proportion higher than 10% (Fig. 2b).

### Quality control of clean reads

Figure 2c,d shows the quality control graphs of the representative sample after filtering. The single base error per position along the read was under 0.04% for all positions and under 0.03% for the 75% of the positions (Fig. 2c). Distribution of error rate was similar between samples showing an overall low value of error in the sequencing process (Supplementary File 1). The percentage of error rate increased at the end of the reads as the sequencing process progressed indicating the reduction of sequencing reagents in the reaction.

The GC content distribution (Fig. 2d) showed similar values between G/C and A/T throughout the sequencing of each read showing the expected profile for non-stranded libraries. After filtering, all samples obtained a Phred quality score above 20 in at least 95.14% of their positions and a Phred quality score of 30 in at least 88.21% of the positions (Supplementary File 1). The Q20 and Q30 average value of the 46 libraries were 96.94% and 91.70%, respectively. Overall results of filtering and quality of clean reads indicated that the sequencing progressed adequately composing a high-quality sequencing dataset.

### Transcriptome reconstruction

**Fig. 3:**
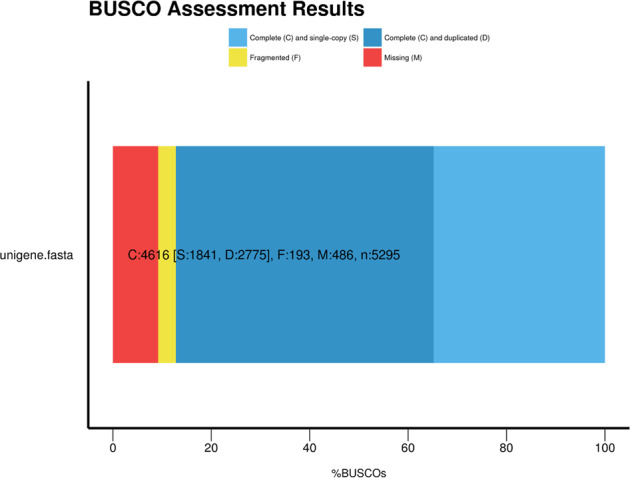
Assembled transcriptome quality. Graphical representation of BUSCO scores of the *O. vulgaris* paralarvae transcriptome: C:87.2% [S:34.8%, D:52.4%], F:3.6%, M:9.2%; n:5295 - Mollusca Odb10 database.

Clean reads were *de novo* assembled by Trinity to obtain the assembled transcriptome. Contigs with a low number of mapped reads (less than 10 reads by default) were filtered out using Corset^47^. After that, hierarchical clustering was performed to reduce redundancies. In order to obtain the best representation of a gene, the longest transcript was selected within each cluster (unigene). BUSCO tool v.3.0.2^56^ was used to assess assembly completeness and annotation quality with the lineage dataset Mollusca Odb10 as reference. Results revealed that 87.2% of the core genes were complete, 3.6% were present but fragmented, and an additional 9.2% were missing. Among complete genes, 52.4% were marked by BUSCO as duplicated (Fig. 3). This transcript redundancy resulted from the high number of biological replicates and developmental stages under different rearing conditions and infection trials used for assembly construction^57^.

Table 1 shows the distribution of sequences by length fragment. The number of unigenes (426515) was slightly lower than the number of transcripts (426736). The higher difference in the abundance of sequences corresponded to the length fragment ranging from 200 to 500 bp. Half of total transcripts and unigenes length between 200 and 500 bp, the 27% of the total corresponded to sequences ranging from 500 to 1 kb, the length fragment up to 2 kb accounted for the 11.9% of the sequences and the 7.3% of the total had a length higher than 2 kb. Metrics on number of base pairs were equal for transcripts and unigenes, being the minimum and maximum length observed 201 bp and 38516 bp long, respectively.

**Table 1:** Number of transcripts and unigenes classified by length intervals and length distribution.

	Transcripts	Unigenes
**Number of sequences:**		
200-500 bp	226553	226334
500-1k bp	117727	117726
1k-2k bp	50903	50902
>2k bp	31553	31553
Total	426736	426515
**Number of base pairs:**		
Minimum length	201	201
Mean length	797	797
Median length	475	475
Maximum length	38516	38516
N50	1139	1139
N90	344	344
Total	340093823	340038556

### Functional annotation

**Fig. 4:**
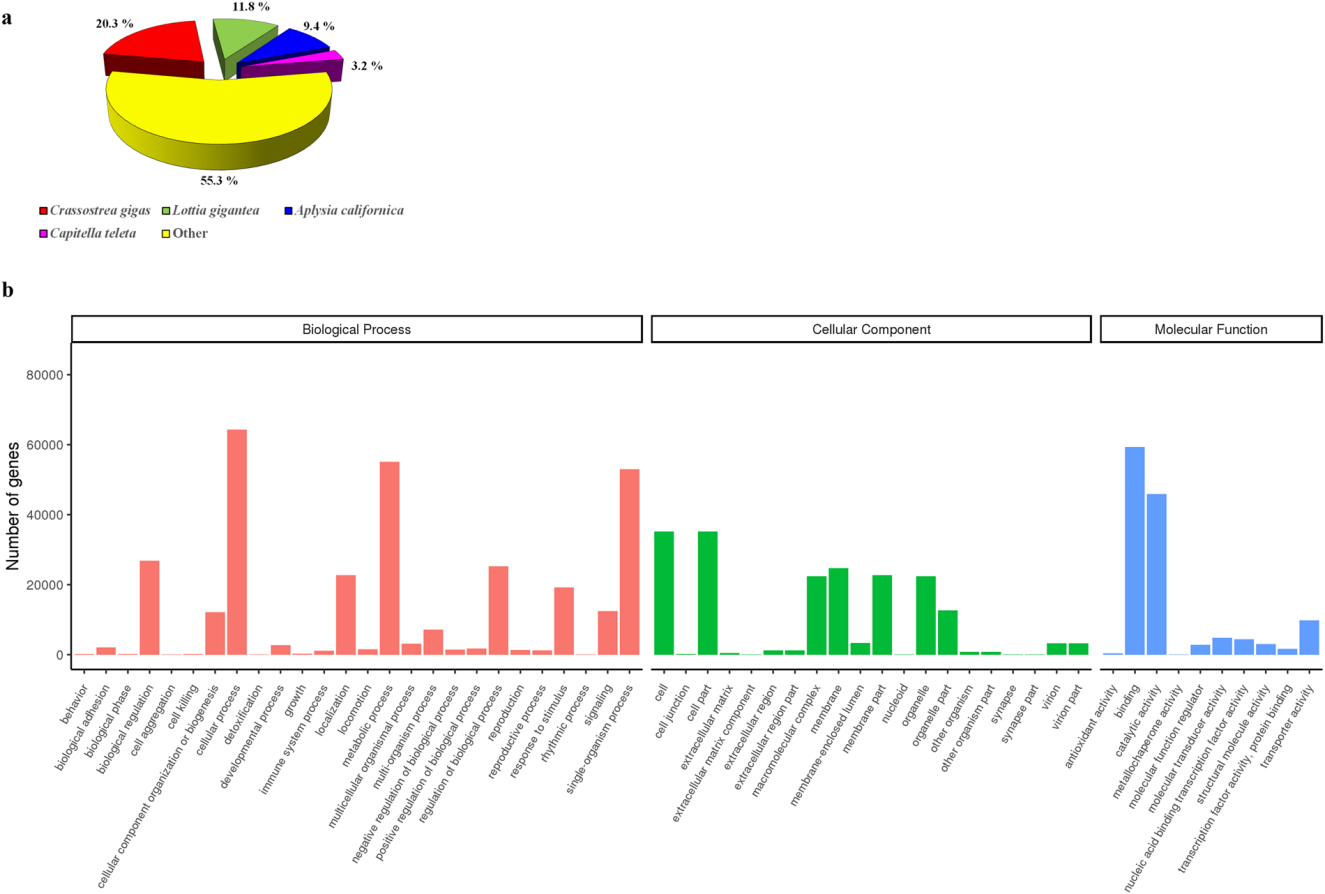
Classification of annotated unigenes. (**a**) Percentage of species similarity based on Nr annotation. (**b**) Number of unigenes successfully annotated into GO Database and grouped into three main GO domains: Biological Process (BP), Cellular Component (CC), and Molecular Function (MF).

Unigenes were functionally annotated in seven databases (Table 2). A total of 192189 unigenes were successfully annotated in at least one database, representing 45% of total unigenes. 0.75% of unigenes (3216) was annotated in all databases. The database with higher annotation proportion was Nt with 35.2% (150149) of total unigenes annotated followed by GO, Nr and Pfam databases with a percentage of annotation of 26.63% (113619), 26.57% (113338) and 26.47% (112932), respectively. A total of 92701 unigenes were annotated in Swiss-Prot (21.73%), 48550 were annotated in KOG (11.38%) and 5572 were annotated in KO (1.3%). Figure 4a shows the percentage of species similarity of successfully annotated unigenes into the Nr database. A total of 41.5% of total unigenes had similarity to different species of molluscs, including the bivalve *Crassostrea gigas* (20.3%) and the gastropods *Lottia gigantea* and *Aplysia californica*. The number of unigenes categorized into the three main categories of GO, Biological Process (BP), Cellular Component (CC), and Molecular Function (MF) is represented in Fig. 4b. Regarding Biological process the terms with higher number of genes are, cellular process, metabolic process, single-organism process and biological regulation. General terms related to the cell (cell and cell part), membrane (membrane and membrane part) and organelle (organelle and organelle part) were the most represented into the Cellular component category. Binding and catalytic activity were the subterms with higher number of unigenes annotated among the Molecular function category.

**Table 2:** Total number and percentage of unigenes successfully annotated in each database.

	Number of Unigenes	Percentage (%)
Annotated in Nr	113338	26.57
Annotated in Nt	150149	35.2
Annotated in KO	5572	1.3
Annotated in Swiss-Prot	92701	21.73
Annotated in Pfam	112932	26.47
Annotated in GO	113619	26.63
Annotated in KOG	48550	11.38
Annotated in all Databases	3216	0.75
Annotated in at least one Database	192189	45.06
Total Unigenes	426515	100

### Gene expression abundance analysis

Reads corresponding to each biological replicate and condition were mapped to the *de novo* assembled transcriptome to estimate unigene abundance using RSEM software. Positional biases generated by the specific protocol of non-stranded library construction were considered in the analysis and final assignment was based on the probability of each read corresponding to each unigene in an iterative process following the maximum likelihood estimation method.

Percentage of mapping for each sample is displayed in Supplementary File 1. The average of mapping, considering the 46 libraries was 70.98% ranging from 64.53% of mapped reads in embryo pooled sample (EMB) to 73.7% in one biological replicate of newly hatched paralarvae (OP_0_5).

**Fig. 5:**
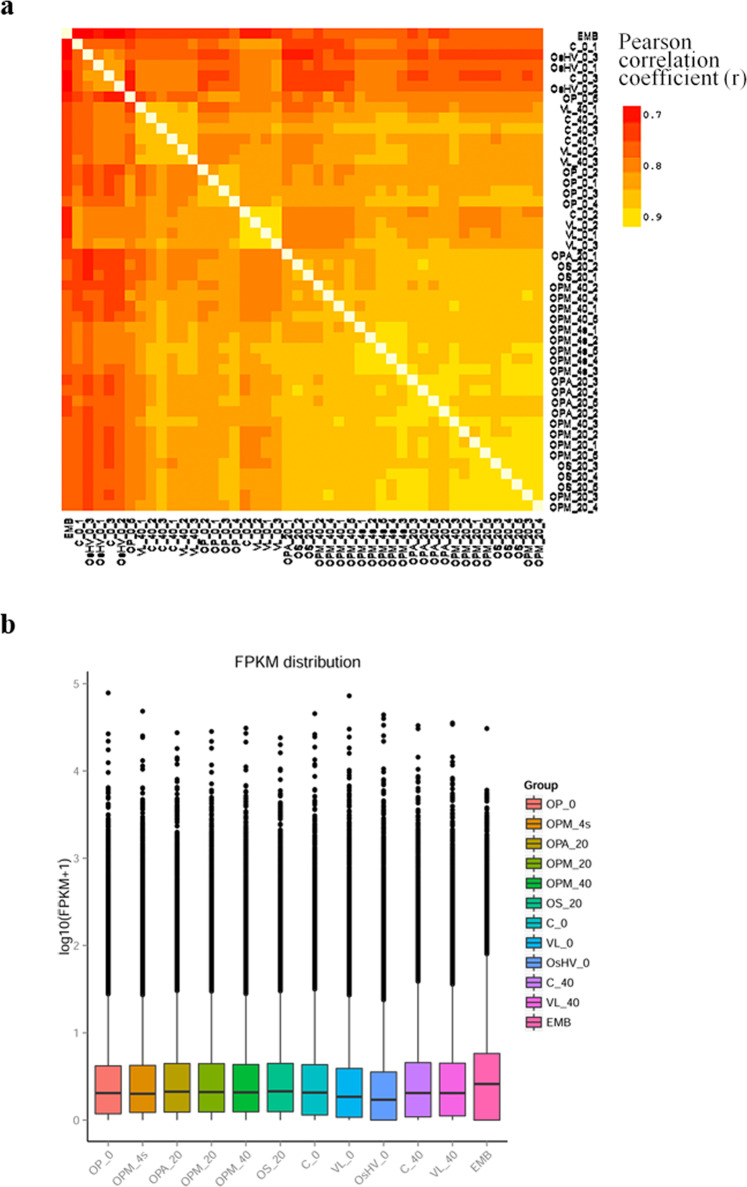
Sample correlation and gene expression level of experimental conditions. (**a**) Scatter diagram of pairwise correlation between samples (Pearson coefficient). (**b**) Box plot of log10(FPKM + 1) per sample group.

Unigene abundance level was normalized considering the depth of sequencing and the length of the transcript using the metric FPKM (Fragments Per Kilobase of transcript sequence per Millions base pairs sequenced). The correlation matrix between samples showed a good similarity between samples and replicates (Fig. 5a). The Pearson correlation values were above 0.7 for all sample pair combinations. The highest values of inter-sample correlation were obtained between samples at 10, 20 and 40 dph, whereas embryo and samples from the infection trials showed the lowest correlation compared to the other conditions.

The distribution of gene expression per condition considering the corresponding biological replicates is represented in a box plot as maximum value, upper quartile, median value and lower quartile of log-transformed data (log10(FPKM + 1)) (Fig. 5b). Similar levels of expression with comparable FPKM were observed between conditions. For each experimental condition, the distribution of expression showed a good repeatability between biological replicates. The median expression value in infection trial samples at 0 dph were slightly lower compared to other conditions and higher variability in expression was observed in embryo sample after overall comparison.

## Supplementary information

Supplementary File 1

## Data Availability

No custom code was used to generate or process the data described in the manuscript.
